# The formetric TRACER index: a valid measure of aesthetic deformity in adolescent idiopathic scoliosis

**DOI:** 10.1186/1748-7161-8-S2-O28

**Published:** 2013-09-18

**Authors:** Francesco Negrini, Fabio Zaina, Stefano Negrini

**Affiliations:** 1 Università Vita-Salute San Raffaele, Milan, Italy

## Background

The gold standard of scoliosis evaluation-Cobb angle measurement-is a valuable tool for assessing spinal deformity and making therapeutic decisions[1,2] but is not fully able to describe the trunk deformity (aesthetics), which is a main concern of patients. Posture, deformity of other bones and soft tissues could be other determinants of the aesthetic impairment due to scoliosis. We do not currently have a commonly accepted measurement of aesthetic impairment due to scoliosis.

## Purpose

The goal of this study was to find a parametric measure to describe aesthetic impairment due to scoliosis. Design: Cross-sectional study. Population: 119 adolescent idiopathic scoliosis (AIS) patients (10.0% males, age 13.9±2.3, 25.4±10.9° Cobb). The sample was randomly divided into two subgroups.

## Methods

Repeated formetric evaluations and simultaneous pictures of the back were performed. An online questionnaire with the photos was completed by 41 laypeople with no scoliosis experience (53.6% males, age 30.3±13.3), who judged the photos on a 0-3 Likert scale (0 normal, 3 highly asymmetric). Statistics: On the first subgroup, we performed a stepwise forward regression technique considering as dependent variable the median of the results of the questionnaire, and as independent variable formetric, clinical and radiological parameters. On the second group, we checked the correlation between the index found (TRACER) and the median of the results of the questionnaire (Spearman’s Rho). Finally, we checked the test-retest repeatability (Spearman’s Rho), concurrent (Spearman’s Rho) and diagnostic (T-test) validity of the index we found.

## Results

The following formula was found: TRACER= [0.726+ (CobbMax*0,018)+(SurfaceRotation*0.037)]/3*100. This TRACER index (0-100) was well-correlated (Rho=0.414, p<0.01) with the median of the questionnaire results in the second group; it was also highly repeatable (Rho=0.922), strongly correlated to Cobb degrees (Rho=0.838), mildly correlated to TRACE index [3] (Rho=0.376) and distinguished pathological and healthy subjects (p<0.01).

## Conclusions and discussion

The TRACER index could be used to measure aesthetic deformity in AIS patients; however, further studies are needed to investigate its role in the conservative and surgical treatment of scoliosis.

## References

[B1] Negrini S Grivas TB Kotwicki T Maruyama T Rigo M Weiss HR Why do we treat adolescent idiopathic scoliosis? What we want to obtain and to avoid for our patients SOSORT 2005 Consensus paper. Scoliosis 2006 1 4 10.1186/1748-7161-1-4 PMC1475888 16759352

[B2] Donaldson S Stephens D Howard A Alman B Narayanan U Wright JG Surgical decision making in adolescent idiopathic scoliosis Spine 2007 32 14 1526 1532 10.1097/BRS.0b013e318067dc75 17572623

[B3] Zaina F Negrini S Atanasio S TRACE (Trunk Aesthetic Clinical Evaluation), a routine clinical tool to evaluate aesthetics in scoliosis patients: development from the Aesthetic Index (AI) and repeatability Scoliosis 2009 4 1 3 10.1186/1748-7161-4-3 19154604 PMC2654427

